# Analysis of relative genes expression and mutation of *pstB* and *efpA* efflux pumps in *Mycobacterium simiae* isolates from suspected tuberculosis patients by using quantitative Real-time PCR

**DOI:** 10.1186/s12866-025-03843-6

**Published:** 2025-03-17

**Authors:** Mohammad Hashemzadeh, Maryam Hasanvand, Effat Abbasi Montazeri

**Affiliations:** 1 Infectious and Tropical Diseases Research Center, Health Research Institute, Ahvaz Jundishapur University of Medical Sciences, Ahvaz, Iran; 2 Department of Microbiology, Faculty of Medicine, Ahvaz Jundishapur University of Medical Sciences, Ahvaz, Iran

**Keywords:** *Mycobacterium simiae*, *pstB efpA*, Efflux pumps, Real-Time PCR

## Abstract

**Background:**

*Mycobacterium simiae* is commonly found in people with weak immune systems such as the elderly and people with tuberculosis and other lung diseases. The aim of this study is the epidemiology of *M. simiae* infections in Iran and the world, therefore, in this study, analysis of relative gene expression and mutation of *pstB* and *efpA* efflux pumps in *Mycobacterium simiae* isolates from suspected tuberculosis patients by using Real-time quantitative PCR. Sixty-five sputa samples of suspected tuberculosis specimens were collected. The identification of NTM Species by PCR sequencing and determining drug sensitivity by micro broth dilution method. The investigate mutations in *pstB* and *efpA* efflux pump genes using the PCR-sequencing method. Comparative evaluation of the replication efficiency of internal control gene 16SrDNA and target genes *pstB* and *efpA*.

**Results:**

In total, 15 isolates of *M. simiae* were evaluated and a drug sensitivity test was performed for them against the antibiotic ethambutol, moxifloxacin, clarithromycin, and linezolid. The highest resistance to moxifloxacin and linezolid antibiotics and the lowest resistance to clarithromycin antibiotics were observed. The results of expression levels of two efflux *PstB* and *efpA* showed that there was a significant difference in the expression level of the *efpA* efflux pump gene in *M. simiae* three-resistant and double-resistant isolates compared to the sensitive group and the standard.

**Conclusions:**

The high level of antibiotic resistance In *M. simiae* isolates is an alarm and monitoring all factors related to antibiotic resistance, including efflux pumps, is an important research topic.

## Background

The Mycobacterium genus includes important species such as *Mycobacterium tuberculosis complex* causing tuberculosis and non-tuberculous mycobacteria (NTM) [1]. Non-tuberculosis mycobacteria are mostly environmental bacteria and are among the opportunistic pathogens that can cause disease in various human and animal organs (Conyers et al,2024). This last mycobacterial group has a great diversity in pathogenicity, adaptation to the environment, pathogenicity, drug response, and growth characteristics [2]. Transmission of these mycobacteria from human to human rarely occurs, however, if this transmission occurs, these organisms can lead to serious complications [3]. Accordingly, NTMs are classified into fast-growing and three slow-growing categories. *Mycobacterium simiae* (*M. simiae*) a slow-growing mycobacterium can cause disease in people with weak immune systems such as the elderly and people with tuberculosis and other lung diseases. *M. simiae* is widely dispersed in the environment and can easily tolerate low temperatures, especially in abundant drinking water [4]. According to the culture characteristics, and biochemical behavior, *M. simiae* strains can be classified into 2 subspecies: serotype 1 strains that produce urease and are variable in the production of nicotinamidase and pyrazinamidase. In contrast, serotype 2 strains only produce urease [5]. Pharmacological treatment of pulmonary and diffuse diseases associated with NTM includes a multidrug regimen containing a macrolide. Clarithromycin is a key drug in the treatment of diseases caused by other mycobacteria than *M. tuberculosis* Complex is mentioned [5]. Clarithromycin is one of the main drugs in treating patients with disease caused by NTMs. It is the only drug recommended by the Institute of Laboratory and Clinical Standards for drug sensitivity tests of NTM [6]. Also, moxifloxacin and trimethoprim-sulfamethoxazole can be used in the treatment of diseases caused by *M. simiae* [6]. Reports from Iran and other parts of the world show that the prevalence of drug resistance among clinical isolates of *M. simiae* is increasing and it is considered an alarm for the treatment of infections related to this species [6, 7]. *M. simiae*, the innate antibiotic resistance is attributed to its lipid-rich cell wall, which is particularly associated with efflux pumps that act as drug carriers. Many drugs are expelled from the cell through these efflux pumps or channels [8, 9]. Purine proteins are also transported out. The efflux pump systems can be classified into five categories:1- ATP-binding cassette (ABC) superfamily, 2- major facilitator (MFS) superfamily, 3- extrusion family multidrug and toxic compounds (MATE), 4- resistance-nodule cell division (RND) superfamily, and 5- small multidrug resistance (SMR) family [10]. Overexpression of efflux pumps increases antibiotic resistance, which is mostly due to mutations in regulatory genes. [11] *pstB* belongs to the superfamily (MFS) and was identified *in M. smegmatis* strain. Its excessive expression causes resistance to ciprofloxacin [12]. *efpA* is a gene encoding the MFS efflux pump of the QacA family [13]. Interestingly, genome microarray analysis of mycobacterial genes showed that *efpA* expression was increased in the presence of isoniazid [14]. It has been shown that *efpA* gene deletion in *M. smegmatis* leads to increased sensitivity to cationic dyes and fluoroquinolones. Deletion of the *efpA* homolog in *Mycobacterium* led to a two-fold increase in sensitivity to ethidium bromide, gentamicin, and fluoroquinolones and an eight-fold increase in sensitivity to acriflavine [15]. Unexpectedly, the mutant also showed a four-fold reduction in sensitivity to rifamycin and chloramphenicol and a two-fold reduction in sensitivity to isoniazid and erythromycin compared to the wild-type (Adhikrao et al,2024). Therefore, due to many reasons such as lack of correct identification, lower prevalence compared to tuberculosis, casting a shadow of tuberculosis in endemic areas of tuberculosis, limited studies specifically related to drug resistance, the status of efflux pumps, and epidemiology of *M. simiae* infections in Iran and the world, this study aims to elucidate the analysis of expression and detection of mutations of *pstB* and *efpA* efflux pumps by suing Real-time PCR and detection of antibiotic resistance in *M. simiae* isolates by using of Minimal Inhibitory Concentration (MIC).

## Methods

### Clinical specimens preparing

Sixty-five sputa samples of suspected tuberculosis patients were collected from referred to ten referral hospitals in Ahvaz City, southwest Iran, from the beginning to the end of 2023. The 65 sputum samples from tuberculosis patients were collected from the Ahvaz Tuberculosis Reference Center in southwest Iran. The two sentences say similar things. Combine them into one. 65 suspected *M. simiae* isolates were tested by conventional biochemical phenotypic tests [16].

### DNA extraction

The mycobacterial isolates grown on LJ medium were used for extraction of genomic DNA using DNA extraction QIAamp Mini Kit (Qiagen, Hilden, Germany), according to the manufacturer’s guidelines. The absorbance ratios of 260/280 nm and 230/260 nm were read for the samples. An elution buffer was used as a blank for the nanodrop device. RNA samples with a ratio of 230/280, which were in the range of 1/8–2.2, were used for cDNA synthesis. After that, they were kept in a freezer at -80 °C until cDNA synthesis.

### Identification of NTM Species by PCR sequencing

For NTM molecular identification, a 750-bp fragment of the *rpoB* gene was amplified using MycoF and MycoR primers [17]. The amplified PCR products for each isolate were purified with the Gene JETTM Gel Extraction Kit (Sina clon, Iran), as per the manufacturer’s guidelines. An ABI PRISM 7500 Sequence Detection System (Applied Biosystems, USA) was used to determine the sequences of the products. The product of the *rpoB* gene for each isolate was examined using BLAST, available pertinent sequences of NTM recovered from the GenBank database, using the MEGA7 program [18].

### Determining drug sensitivity by micro broth dilution method

In this study, the MIC of the clarithromycin, linezolid, ethambutol and moxifloxacin antibiotics in *M. simiae* isolates was evaluated using the micro broth dilution method. Middlebrook 7H9 (Conda, India) culture medium was used to perform this method. After preparing this medium, 100 µL of medium was divided into 96 plates. To prepare antibiotic dilutions, the drugs were first prepared with distilled water or another suitable solvent (according to the instructions of the drug manufacturer and according to the potency of each antibiotic) in different dilutions (the concentration of the drug is determined according to the drug used). Then, by diluting these antibiotics in microplate wells, the desired concentration range for each antibiotic was prepared according to CLSI guidelines. It should be noted that to prepare antibiotic dilutions, 200 µl of Middlebrook 7H9 medium, including the desired antibiotic, was inoculated in the first well, and 100 µl of the solution was transferred from the first to the second well and from the second to the third well. In this way, it continued until the witness wells. In this way, the drug concentration in each well will be diluted twice compared to the previous well. The MIC, expressed in mg/L, is the lowest concentration that inhibits visual growth. Mycobacterium tuberculosis H37Rv ATCC 27294 is used as the reference strain and its targeted MIC values are within the range > 16 for Clarithromycin, > 2 for Moxifloxacin, > 4 L for Moxifloxacin and > 16 for Linezolid [19].

### Investigating mutations in *pstB* and *efpA* efflux pump genes using PCR method

The primers used for *pstB* and *efpA* genes in this study were designed with Primer 3 software (Table 1).

**Table 1:** Primers used for amplification of target genes

target sequence	Primers	( ˈ 3 ˈ5)	Bp	Ref
***PstB***	Forward	F: 5'- GGTAAAGGACGCAGCTACAAG -3'	bp 311	This study
	Reverse	R: 5' ATAACTCTCCCCATTGCAGGT -3'		
***EfpA***	Forward Reverse	F: 5' AGGAACACTTGCTCTTTGAGCC -3' R: 5' CAAATCACTCCCAAGCTGGC -3'	bp 209	This study

After the identification of *M. simiae* isolates, the isolates were prepared to evaluate the expression level of the efflux pump genes *pstB* and *efpA* for RNA extraction. To extract RNA in this study, RNA isolation kit-RNX-Plus (Sinaclon-Iran) was used according to the instructions of the company. After transfer, the samples were quantitatively and qualitatively evaluated using a nanodrop device (Thermo Scientific, Waltham, MA). The absorbance ratios of 260/280 nm and 230/260 nm were read for the samples. An elution buffer was used as a blank for the nanodrop device. RNA samples with a ratio of 230/280, which were in the range of 2.2–1.8, were used for cDNA synthesis. After that, they were kept in a freezer at -80 °C until cDNA synthesis.

### Treatment of extracted samples with DNase

After the quantitative and qualitative assessment of RNA, the samples were treated with a DNase kit (Sinaclon-Bioscience, Iran) to remove any possible contamination with DNA. The protocol used to treat the samples with Dnase enzyme was carried out according to the manufacturer's instructions.

### cDNA synthesis

cDNA synthesis was carried out using the cDNA synthesis kit of the company (Sinaclon-Iran) according to its instructions.

### Real-time PCR reaction

The real-time PCR method was used to quantitatively check the expression of target genes. The real-time PCR reaction was performed using specific primers of 16SrDNA gene as normalizer and *pstB* and *efpA* genes (the sequence of primers is listed in Table 2) and according to the protocol of the SYBR1 Green high ROX Master Mix kit (Ampliqon, UK).

**Table 2:** Efflux pump primers used in Real-Time PCR

target sequence	primers	( ˈ 3 ˈ5)	Bp	Ref
***PstB***	Forward	F: 5'- GGTAAAGGACGCAGCTACAAG -3'	bp 311	This study
	Reverse	R: 5' ATAACTCTCCCCATTGCAGGT -3'		
***EfpA***	Forward Reverse	F: 5' AGGAACACTTGCTCTTTGAGCC -3' R: 5' CAAATCACTCCCAAGCTGGC -3'	bp 206	This study
***16SrDNA***	Forward Reverse	F: 5' CTTAACACATCGAAC -3' R: 5' GTATCTCAGTCCCAGTGTG -3'	bp 278	**7**

To check the expression of each gene, a mixture of SYBR Green high ROX Master Mix, sterile distilled water, and forward and reverse primers were prepared in a 1.5 ml microtube free of DNase and RNase. Then the contents of this microtube were divided into special strips for the Real-Time PCR device according to the number of reactions to be performed in one step. Then 3 µl of cDNA related to the standard sample and the investigated samples were added to the strips to check the expression of the desired genes and finally the reaction strips were placed in the device.

### Comparative evaluation of the replication efficiency of internal control gene *16SrDNA* and target genes *pstB* and *efpA*

Considering the selection of the comparative method of ΔΔCt gene expression in this study, it was necessary to evaluate the efficiency of internal control gene amplification and target genes. To use the above formula to be valid, the replication efficiency of the target gene and the internal control gene must be almost equal. For this purpose, the log standard curve of diluted standard values ​​of each gene was drawn against CT values. In this way, a sample of cDNA products was selected and dilutions of 100, 10, 1, 0.1, and 0.01 were prepared for each gene. The real-time PCR reaction was performed on the samples as previously described, repeating twice for each sample and each gene (target and calibrator). If the efficiency of target and reference genes is the same, the slope of the graph should be almost equal.

### Comparative analysis of gene expression

The results were determined using the comparative method of ΔΔCt and using step oneTM software v.2.0.2 and the fold changes of the relative expression of mRNAs were calculated using the 2^−ΔΔCt^ method compared to the standard H37Rv strain. In this study, results equal to one and less than one indicate that the gene expression level is similar to the standard strain, expression levels higher than 1 as increased expression and increased expression more than 4 times or more in the expression of target genes are used as overexpression.

### Statistical analysis

The Fisher exact probability method was used when the theoretical frequency of cells was < 5. The Cochran-Armitage trend test was used to analyze the detection rate of pathogenic bacteria and the rate of drug resistance. The statistical analysis was performed with SPSS 22 software. *P* values < 0.05 were considered statistically significant.

## Results

### Nontuberculous mycobacterium isolates investigated

In this study, during one year, the clinical isolates of NTM from the regional TB reference laboratory of Khuzestan province, based on the initial tests in terms of pigment production and growth rate. They were separated, collected regularly (weekly) with the arrangements made and entered into the study. In the present study, 65 clinical specimens from the regional tuberculosis reference laboratory of Khuzestan province, which had sufficient clinical information, were examined and evaluated. These isolates were initially suspected of NTM in tuberculosis laboratories due to reasons such as treatment failure and doctor's diagnosis. In this study, the identity of slow-growing pigmented *Mycobacterium* isolates was determined with the aim of isolating and identifying *M. simiae* isolates from among the collected isolates. Then *M. simiae* isolates were transferred to the molecular laboratory of the Department of Microbiology of the Faculty of Medicine to perform molecular tests.

### Molecular confirmation of *M. simiae* species using *rpoB* gene sequence and sequencing

Among the 30 isolates suspected to be *M. simiae*, the *rpoB* gene was investigated using PCR and sequencing methods.

### Identification of isolates based on *rpoB* gene sequence

MEGA and Blastn software were used for species identification. The *rpoB* sequence of the reference *M. simiae* strain and other species close to it (slow-growing and photochromogenic non-tuberculous mycobacteria) were entered into this software in the international database, and the *rpoB* sequence of clinical isolates was entered into the software and manually and by using the software, the process of arranging the sequences with the prepared database was done. Among the 30 isolates suspected to be *M. simiae*, 15 (50%) isolates were confirmed and identified as *M. simiae*. Other isolates identified by this gene include 10 (33.33%) *M. kansasii* 1 (3.33%) *M. intracellulare*, 2 (6.66%) M*. szulgai* and 2 (6.66%) *M. asiaticum*, which were excluded from the study. 15 confirmed isolates of *M. simiae* were included in the next stages of the research to perform antibiotic resistance tests and molecular methods. Demographic information of 15 M*. simiae* isolates is shown in Table 3. 11 (73.33%) samples were taken from men and 4 (26.66%) samples were taken from women. Ten (66.66) sputum samples, 2 (13.33) BAL samples, and three (14.34) BAL and sputum samples were combined.

**Table 3:** Socio-demographic characteristics of tuberculosis patients infected with *M. simiae* isolates

**Variables**		**N**	**%**
**Sex**	**Female**	12	40
	**Male**	18	60
**Age (years)**	**18–30**	5	Jan-66
	**31–49**	12	40
	**50**	13	34.33
**BMI (kg/m2)**	** < 18.5**	7	2.33
	**18.5–22.9**	14	46.66
	**23**	9	30
**Chronic diseases**	**hypertension**	12	40
	**diabetes**	14	46.66
	**asthma**	4	1.33
**Recurrent TB**	**Yes**	10	33.33
	**No**	20	66.66
**Patient status**	**MDR-TB**	18	60
	**Non-MDRTB**	12	40

### Phenotypic test results of micro broth dilution of drug resistance in *M. simiae* isolates

In this study, phenotypic drug resistance tests by the MIC.in 15 isolates of *M. simiae* were investigated using the micro broth dilution method for moxifloxacin, clarithromycin, ethambutol, and linezolid. The MICs obtained from this research are listed in Table 4. It should be noted that the MIC for each antibiotic was based on the latest CLSI guidelines. Also, the drug resistance profiles of the studied strains are shown in Tables 5 and 6.

**Table 4:** Drug susceptibility profile of M. simiae to antibiotics

	**Isolate**	MOX*	CLR*	LIN*	EB*
		**(µg/mL)**	**(µg/mL)**	**(µg/mL)**	**(µg/mL)**
**1**	**NTM10**	4	2	32	8
**2**	**NTM20**	8	2	32	8
**3**	**NTM28**	4	2	64	64
**4**	**NTM44**	16	2	32	64
**5**	**NTM100**	4	2	64	4
**6**	**NTM102**	4	2	32	2
**7**	**NTM315**	8	2	64	4
**8**	**NTM342**	32	2	32	2
**9**	**NTM23**	32	32	64	32
**10**	**NTM51**	2	2	4	2
**11**	**NTM54**	2	8	4	2
**12**	**NTM50**	2	8	4	4
**13**	**NTM49**	2	4	8	2
**14**	**NTM221**	2	4	4	2
**15**	**NTM115**	2	4	4	2

*MOX* Moxifloxacin, *CLR* Claritromycin, *LIN* Linezolid, *INH* Isoniaside, *EB* Ethambutol

**Table 5:** Minimum inhibitory concentration for each drug

Antimycobacterial Agent	MIC Indicating Resistance (µg /mL)
Clarithromycin	> 16
Moxifloxacin	> 2
Ethambutol	> 4
Linezolid	> 16

**Table 6:** Drug susceptibility profile of M. simiae to antibiotics

	**Isolat**	MOX*	CLR*	LIN*	EB*
		**(µg/mL)**	**(µg/mL)**	**(µg/mL)**	**(µg/mL)**
**1**	**NTM10**	4	2	32	8
**2**	**NTM20**	8	2	32	8
**3**	**NTM28**	4	2	64	64
**4**	**NTM44**	16	2	32	64
**5**	**NTM100**	4	2	64	4
**6**	**NTM102**	4	2	32	2
**7**	**NTM315**	8	2	64	4
**8**	**NTM342**	32	2	32	2
**9**	**NTM23**	32	32	64	32
**10**	**NTM51**	2	2	4	2
**11**	**NTM54**	2	8	4	2
**12**	**NTM50**	2	8	4	4
**13**	**NTM49**	2	4	8	2
**14**	**NTM221**	2	4	4	2
**15**	**NTM115**	2	4	4	2

*MOX* Moxifloxacin, *CLR* Claritromycin, *LIN* Linezolid, *INH* Isoniaside, *EB* Ethambutol

### Results of drug resistance test

**Fig. 1:**
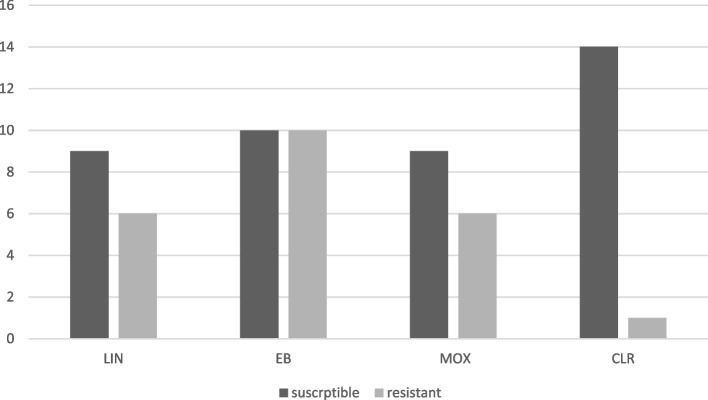
Frequency of susceptible and resistant isolates of *M. simiae*

Out of 15 isolates of *M. simiae* identified in this study, 1 (0.06) isolate was resistant to all four drugs. Also, 6 (40%) isolates of *M. simiae* were sensitive to all antibiotics. The highest resistance to moxifloxacin and linezolid antibiotics and the lowest resistance to clarithromycin antibiotics were observed. Also, the frequency of susceptible and resistant isolates of *M. simiae* is shown in Fig. 1.

### Drawing a standard curve to calculate the efficiency of the desired genes

**Fig. 2:**
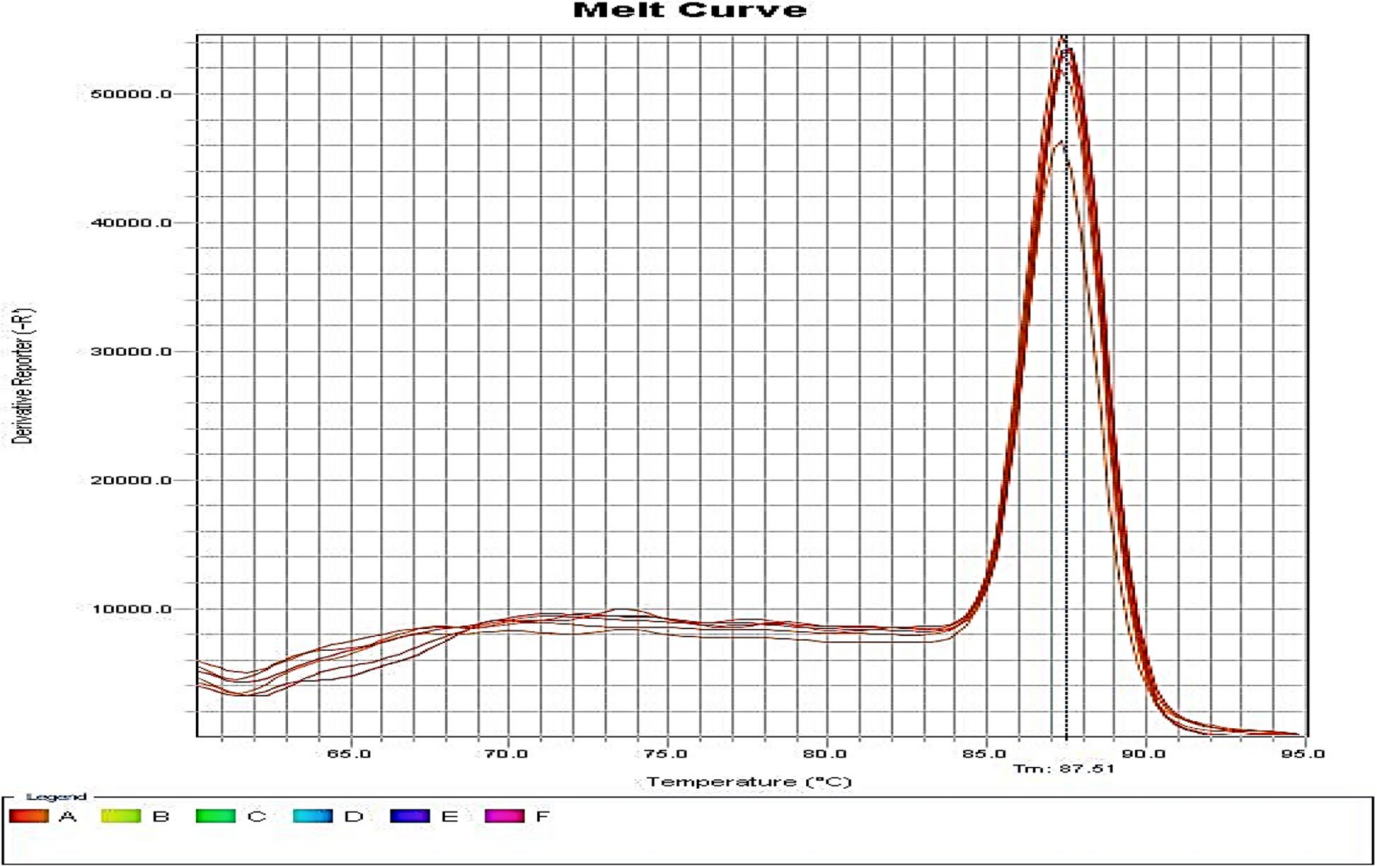
Melting curve diagram of 16SrDNA gene

**Fig. 3:**
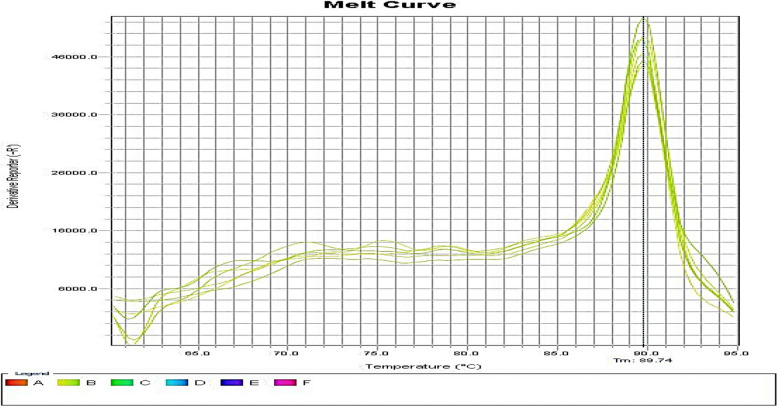
Melting curve diagram of *efpA* gene

**Fig. 4:**
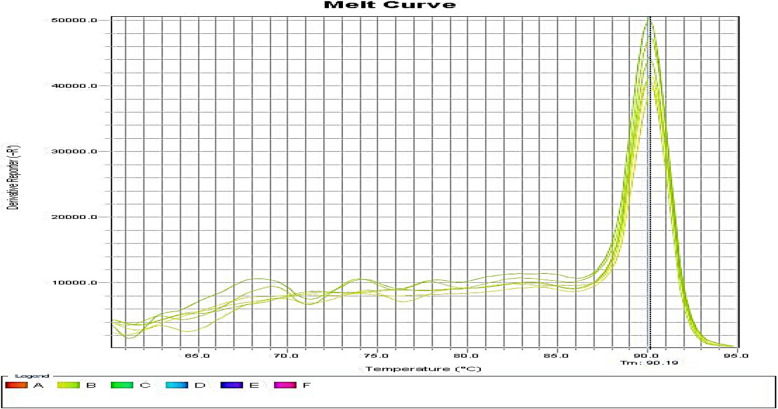
Melting curve diagram of *PstB* gene

Evaluation of *16SrDNA* gene amplification efficiency was done as an internal control gene and *efpA* and *PstB* genes as target genes.The standard log curve was drawn against the CT values ​​which were within the acceptable range.standards and pump efflux. Figs. 2, 3, 4. Also, the melting temperatures of the *PstB* and *efpA* and *16SrDNA* genes.

### Quantification of the drug resistance levels in the presence of efflux pumps

We quantified the drug resistance levels of the *M. simiae* isolates to the antibiotics. For this purpose, the MICs of the antibiotics and efflux pumps were determined for each isolate and the results are presented in Table 7.

**Table 7:** **|**MIC of antibiotics and efflux for the *M. simiae* isolates

Isolate	MOX*	CLR*	LIN*	EB*	*EfpA*	*PstB*	Fold Change	Fold Change
	**(µg/mL)**	**(µg/mL)**	**(µg/mL)**	**(µg/mL)**			***efpA*** gene	**PstB**
**NTM10**	4(R)	2	32(R)	8(R)	3.7	2	3.7	2
**NTM20**	8(R)	2	32(R)	8(R)	1.91	3.15	3.15	1.9
**NTM28**	4(R)	2	64(R)	64(R)	**5.6**	0.91	5.6	0.91
**NTM44**	16(R)	2	32(R)	64(R)	**5.8**	0.88	5.8	0.88
**NTM100**	4(R)	2	64(R)	4	**4.1**	1.2	4.1	1.2
**NTM102**	4(R)	2	32(R)	2	**4**	1.11	4	1.11
**NTM315**	8(R)	2	64(R)	4	3.7	1	3.7	1
**NTM342**	32(R)	2	32(R)	2	3.5	2.1	3.5	2.1
**NTM23**	32(R)	32(R)	64(R)	32(R)	0.9	0. 61	0.9	0. 61
**NTM51**	2	2	4	2	1.3	0.32	0.85	0. 72
**NTM54**	2	8	4	2	2	1	1.1	0.9
**NTM50**	2	8	4	4	1.3	3.3	3.7	2
**NTM49**	2	4	8	2	0.9	1.7	3.15	1.9
**NTM221**	2	4	4	2	1.5	1.7	5.6	0.91
**NTM115**	2	4	4	2	1.6	1.3	5.8	0.88
***P* value**							***P* = 0.006**	***P* = 0. 391**

### Resistance levels for moxifloxacin

Out of 15 M*. simiae* isolates, 9 were found to be resistant to Mox. Among the resistant *M. simiae* isolates, 4 M*. simiae* isolates had low-level resistance (R ≥ 4 μg/ml), 2 M*. simiae* isolates had intermediate-level resistance (R ≥ 8 μg/ml), and 3 M*. simiae* isolates had high-level resistance (R ≥ 32 μg/ml). Efflux activity of *efpA* was detected in 4 M*. simiae* isolates. Out of these 4 M*. simiae* isolates, 3 M*. simiae* isolates were low-resistance (R ≥ 4 μg/ml), and one isolate had high-level resistance (R ≥ 16 μg/ml). Efflux activity of *PstB* was not detected.

### Resistance levels for clarithromycin

Out of 15 M*. simiae* isolates, only 1 showed high-level resistance (R ≥ 32 μg/ml) to CLR.

### Resistance levels for linezolid

Out of 15 M*. simiae* isolates, 9 M*. simiae* isolates resistant to LIN presented. All 9 M*. simiae* isolates had high resistance (R ≥ 16–32 μg/ml) to LIN. Efflux activity of *efpA* was detected in 4 M*. simiae* isolates. Efflux activity of *PstB* was not detected.

### Resistance levels for ethambutol

Out of 15 M*. simiae* isolates, 4 M*. simiae* isolates were resistant to EB presented. 3 M*. simiae* isolates of high-resistance (R ≥ 64 μg/ml), 1 M*. simiae* isolates of intermediate-resistance (R ≥ 8 μg/ml) to EB. Efflux activity of *efpA* was detected in 2 M*. simiae* isolates. Efflux activity of *PstB* was not detected.

### Comparison of expression changes of *efpA* and *PstB* efflux pump genes in antibiotic-resistant and sensitive *M. simiae* isolates

The expression levels of two efflux pump genes, *PstB* and *efpA*, in sensitive and resistant isolates (double-resistant, triple-resistant, and quadruple-resistant) compared to the standard and sensitive H37Rv strain were compared using the Kruskal–Wallis test due to the non-normality of the data distribution. To check the expression level of both *PstB* and *efpA* genes, all resistant isolates plus 2 susceptible *M. simiae* isolates were randomly and standardly prepared for RNA extraction, and Real-Time PCR was prepared. The expression level of the *efpA* gene in double-resistance and triple-resistance *M. simiae* isolates was higher than the sensitive and standard *M. simiae* isolates. The results showed that there was a significant difference in the expression level of the *efpA* efflux pump gene in *M. simiae* tri-resistant and double-resistant isolates compared to the sensitive group and the standard strain (*P*-value = 0.006). Also, there was no significant increase in isolate number 23, which was resistant to all four antibiotics mentioned in the study. This lack of increase in resistance can be due to other reasons, such as antibiotic resistance mechanisms, such as mutations in the antibiotic resistance genes. However, no significant difference was observed in the efflux of the *PstB* pump in *M. simiae* isolates with multiple resistance and double resistance compared to the sensitive group and the standard strain (*P*-value = 0.391). Also, according to Table 8, no significant difference was observed between the sensitive group and the standard strain (*P* > 0.05). Next, the expression level of genes for the studied isolates was divided into three groups. The first group had expression ≤ 1, the second group had more expression from 1 to 3.99 and the third group had expression ≤ 4. Overexpression (4 ≤ fold-change) in the *efpA* gene was seen in three-resistant (28 and 44) ​​and double-resistant (100 and 102) isolates of *M. simiae*, with a fold-change of 5.6, 5.8, 4.1, and 4, respectively, and it was not observed in the *PstB* gene.

**Table 8:** MIC of antibiotics significant difference between the sensitive and standard strain

	**Isolate**	MOX*	CLR*	LIN*	EB*	***EfpA***	***PstB***
		**(µg/mL)**	**(µg/mL)**	**(µg/mL)**	**(µg/mL)**		
**1**	**NTM10**	4(R)	2	32(R)	8(R)	3.7	2
**2**	**NTM20**	8(R)	2	32(R)	8(R)	1.91	3.15
**3**	**NTM28**	4(R)	2	64(R)	64(R)	**5.6**	0.91
**4**	**NTM44**	16(R)	2	32(R)	64(R)	**5.8**	0.88
**5**	**NTM100**	4(R)	2	64(R)	4	**4.1**	1.2
**6**	**NTM102**	4(R)	2	32(R)	2	**4**	1.11
**7**	**NTM315**	8(R)	2	64(R)	4	3.7	1
**8**	**NTM342**	32(R)	2	32(R)	2	3.5	2.1
**9**	**NTM23**	32(R)	32(R)	64(R)	32(R)	0.9	0. 61
**10**	**NTM51**	2	2	4	2	1.3	0.32
**11**	**NTM54**	2	8	4	2	2	1
**12**	**NTM50**	2	8	4	4	1.3	3.3
**13**	**NTM49**	2	4	8	2	0.9	1.7
**14**	**NTM221**	2	4	4	2	1.5	1.7
**15**	**NTM115**	2	4	4	2	1.6	1.3

### Evaluating the correlation of expression of two genes to determine the simultaneous expression of *efpA* and *PstB* genes

Based on the results, due to the small sample size and non-normal distribution, Spearman's non-parametric correlation test was used. The test results showed that there is no positive and significant relationship between the expression of both genes. (*P* = 0.512) There is no gene co-expression between *efpA* and *PstB* genes. The co-expression of genes is one of the topics that is of interest to researchers in eukaryotic and prokaryotic genomic and molecular evaluations. The co-expression of genes can indicate that the increase or decrease of each of the co-expressed genes leads to the increase or decrease of the other. In other words, the co-expression of genes indicates the existence of common regulatory mechanisms for their expression. Using Spearman's correlation test, it was determined that the two genes examined in the present study are not expressed the same; That is, the increase or decrease of one of them is not proportional to the increase or decrease of the other gene. Therefore, based on this result, it can be said that *efpA* and *PstB* genes have different mechanisms of gene expression regulation and their expression is independent of each other.

## Discussion

*M. simiae* is a slow-growing, photochromogenic mycobacterium that is pathogenic in humans and animals, primarily affecting immunocompromised individuals, including the elderly and those with tuberculosis (TB) or other pulmonary diseases. In mycobacteria, intrinsic antibiotic resistance is primarily attributed to the low permeability of the cell wall, particularly its complex barrier structure, which synergistically interacts with membrane-associated efflux pumps. Overexpression of multidrug resistance efflux pumps, often resulting from mutations in regulatory genes, contributes to increased bacterial resistance [20].

Limited research on nontuberculous mycobacteria (NTM) remains challenging, as these bacteria are frequently undiagnosed or misdiagnosed, leading to inappropriate treatment regimens. The lack of proper diagnosis, pathogenic potential, and antibiotic resistance have facilitated the spread of resistant NTM species, increasing the risk of systemic and pulmonary infections [21]. Given the scarcity of studies in this area, the present study aimed to determine the frequency and expression of the efflux pump genes *pstB* and *efpA* in drug-resistant M*. simiae* isolates obtained from suspected TB patients referred to the regional TB reference laboratory in Khuzestan Province.

In this study, *M. simiae* was identified in 15 (4.13%) out of 65 NTM isolates, representing a 25% decrease compared to our previous study [6]. The lower prevalence observed may be due to the localized nature of the isolates, as these strains are specific to Khuzestan Province. Regional studies generally report a lower prevalence of *M. simiae* compared to multi-center investigations. In a study by Dezhkhi et al. in Iran, *M. simiae* was the most frequently isolated slow-growing mycobacterium from clinical samples [22]. Similarly, another Iranian study reported a higher prevalence of *M. simiae* than other NTM species [23].

Conversely, studies conducted in India, China, and Spain have identified *M*. *abscessus*, *M. kansasii*, and *Mycobacterium avium complex* (MAC) as the predominant NTM species [24–26]. Variations in NTM prevalence across different regions may be influenced by geographical diversity, the epidemiological status of TB, differences in healthcare infrastructure and diagnostic methodologies, and the prevalence of immunocompromised individuals in each population [27]. A study conducted in Kenya in 2023 reported that among 145 patients with suspected pulmonary infections, *M. simiae* was isolated from 54 cases, all of whom had underlying conditions. These findings highlight the opportunistic pathogenicity of *M. simiae*, particularly in immunocompromised patients [28].

Like many NTM species, *M. simiae* exhibits intrinsic or acquired resistance to first-line TB drugs. The underlying resistance mechanisms include (1) lack of prodrug activation, (2) polymorphisms in drug-activating genes, (3) ADP-ribosylation, and (4) efflux pump activity. Notably, antibiotic susceptibility testing in NTM isolates presents significant challenges, as laboratory susceptibility results often differ from in vivo responses. The 2007 IDSA guidelines recommend a treatment regimen for *M. simiae* based on macrolides in combination with moxifloxacin and clofazimine. However, in immunocompromised individuals, a regimen comprising clarithromycin, ethambutol, and ciprofloxacin is prescribed for disseminated M. simiae infections [19].

This study assessed antibiotic resistance to ethambutol, clarithromycin, linezolid, and moxifloxacin in* M*. *simiae* isolates. Among 15 isolates, one was resistant to all four antibiotics. Resistance to three drugs—moxifloxacin, ethambutol, and linezolid—was observed in four isolates (66.26%), while another four isolates (66.26%) exhibited resistance to moxifloxacin and linezolid. Additionally, six isolates (40%) were susceptible to all tested antibiotics. Overall, nine isolates demonstrated multidrug resistance, highlighting the substantial antibiotic resistance of *M*. *simiae*.

A previous study on drug susceptibility profiles and genetic determinants of resistance in *M. simiae* isolates from regional TB reference laboratories in Iran identified 53 isolates as *M. simiae* based on biochemical tests. Drug susceptibility testing (DST) revealed that all 53 isolates were resistant to isoniazid, rifampin, and clofazimine. Resistance to ethambutol and linezolid was observed in 34 (64%) and 40 (76%) isolates, respectively. The highest susceptibility rates were recorded for amikacin (53 isolates, 100%) and clarithromycin (45 isolates, 85%), followed by moxifloxacin (35 isolates, 66%) [6].

The present study observed the highest resistance to linezolid and moxifloxacin, while clarithromycin showed the lowest resistance. Despite differences in sample size and the number of antibiotics tested, both studies reported similar trends—high susceptibility to clarithromycin and significant resistance to moxifloxacin. Variations in findings may be attributed to differences in bacterial resistance mechanisms, sample size disparities, and the inherent challenges of antibiotic susceptibility testing in NTM isolates. Significantly, laboratory resistance levels do not always correlate with clinical resistance in patients.

A retrospective study by Allou et al. in France (2002–2017) assessed antibiotic resistance in 97 M*. simiae* isolates. Seventy percent of the isolates exhibited limited susceptibility to most antibiotics, except amikacin, fluoroquinolones, and clarithromycin, demonstrating comparatively higher activity [29].

The results of this study differ from our findings, particularly regarding clarithromycin resistance, which was relatively low in our study. Similarly, a study by Hamieh et al. (2018) in Lebanon identified *M. simiae* in 103 isolates from 253 suspected TB patients, with most cases occurring in male patients with a history of smoking. The majority of isolates (97%) were obtained from sputum samples. Treatment regimens commonly included clarithromycin in combination with trimethoprim-sulfamethoxazole, moxifloxacin, and amikacin. Clofazimine was prescribed in only two cases where isolates resisted only one antimicrobial agent. The duration of treatment ranged from 6 to 24 months. While primarily based on IDSA guidelines, trimethoprim-sulfamethoxazole suggests an increasing prevalence of antibiotic resistance in Lebanon, a trend consistent with our findings [5].

This study analysed the efflux pump genes *pstB* and *efpA* expression in drug-resistant *M. simiae* isolates. Initially, mutations associated with these efflux pumps were assessed. *efpA* encodes an efflux pump involved in fluoroquinolone and aminoglycoside resistance, while *pstB* encodes a fluoroquinolone-associated efflux pump. No mutations were detected in these genes. Gene expression analysis revealed that *efpA* expression was significantly higher in antibiotic-resistant *M. simiae* isolates compared to sensitive isolates (*P* = 0.006). However, *pstB* expression did not differ significantly between resistant isolates, sensitive isolates, and the standard strain (*P* = 0.391). Overexpression (fold-change ≤ 4) was detected in the *efpA* gene in four *M. simiae* isolates but was absent in *pstB*.

In 2004, Li et al. investigated efflux pump-mediated intrinsic drug resistance in *M. smegmatis*. Deleting the *efpA* homolog increased bacterial susceptibility to antibiotics while paradoxically reducing susceptibility to rifamycin, isoniazid, and chloramphenicol, resulting in a two- to four-fold increase in the minimum inhibitory concentration (MIC). Additionally, *efpA* deletion increased susceptibility to ethidium bromide, acriflavine, and erythromycin [29].

Another study demonstrated that *efpA* overexpression in *M. smegmatis* enhanced tolerance to multiple first- and second-line TB drugs, including rifampicin, isoniazid, streptomycin, and amikacin. Overexpression of the *efp*,*A* efflux pump led to more than a 180-fold increase in *M. smegmatis* resistance to moxifloxacin, accompanied by reduced uptake of norfloxacin, moxifloxacin and ethidium bromide [29].

The efpA efflux pump is present in both pathogenic and non-pathogenic mycobacteria. In *M. bovis*, which shares 80% sequence similarity with *M. smegmatis efpA*, increased *efpA* expression was associated with an eight-fold increase in tolerance to moxifloxacin, reducing the antimicrobial efficacy of various drugs [30]. A study by Sulami et al. in Iraq analyzed 150 sputum samples from suspected TB patients, assessing resistance to rifampicin, ethambutol, pyrazinamide, isoniazid, and streptomycin. Among the 150 isolates, *M. tuberculosis* overexpressed the *efpA* efflux pump in 23 cases, conferring resistance to ethambutol, pyrazinamide, isoniazid, and streptomycin. The findings raised concerns about *efpA* overexpression and its role in increasing multidrug resistance in M. tuberculosis isolates, potentially compromising TB treatment regimens [31].

A study by Ingen et al. in Germany assessed antibiotic resistance in MAC isolates against first-line TB drugs and other antibiotic classes. Eighty percent of the isolates belonged to MAC. Resistance testing revealed that all isolates were resistant to anti-TB antibiotics, including rifampicin, ethambutol, and isoniazid. The primary role of these drugs in MAC treatment regimens is to prevent macrolide resistance, while their direct antimycobacterial activity is less critical for treatment success. Additionally, 54% of the isolates resisted fluoroquinolones, and 23% exhibited resistance to tetracyclines.

Expression of the *pstB* efflux pump gene was analyzed in MAC isolates. Among isolates resistant to first-line TB drugs, 40% expressed pstB. Furthermore, 33% and 12% of fluoroquinolone- and tetracycline-resistant isolates, respectively, exhibited pstB expression. Unlike the present study, in which *pstB* expression was low in M. simiae isolates, these findings suggest that *pstB* expression is more pronounced in other NTM species [32].

This study identified moxifloxacin and ciprofloxacin as the most effective antibiotics, with 100% susceptibility among isolates. These findings differ from our study, particularly regarding antibiotic resistance patterns. The relatively high resistance observed in *M. simiae* isolates in the present study, compared to Heydarieh's findings, suggests an increasing trend of antibiotic resistance in *M. simiae*. The high prevalence of resistance underscores the urgent need for systematic DST and greater attention to NTM infections within Iran's healthcare system.

The increasing antibiotic resistance observed in *M. simiae* isolates highlights the risk of resistance transmission among mycobacterial strains, potentially leading to higher patient mortality, the need for alternative treatments with more significant side effects, and increased therapeutic failure rates. Addressing these challenges requires prioritizing resistance monitoring and integrating routine susceptibility testing into national health policies. Ultimately, this study aims to provide insights that contribute to answering key research hypotheses and guiding future clinical and epidemiological strategies.

### Limitation

Due to the low financial budget and expensive sequence in Iran, the strains evaluated in the mutation test were not sent for sequencing, and the mutations were obtained using the NCBI website.

## Conclusion

In the conclusion of this study, it can be acknowledged that ignoring any of the NTM isolates in the future will cause concern among doctors and health officials regarding the widespread increase of antibiotic resistance in these isolates in the future. The high level of antibiotic resistance In *M. simiae* isolates is an alarm and monitoring all factors related to antibiotic resistance, including efflux pumps, is an important research topic. Despite such high antibiotic resistance, NTM isolates can no longer be called only environmental bacteria, and taking care of drug resistance is one of the important duties of doctors and researchers.

## Data Availability

No datasets were generated or analysed during the current study.

## Abbreviations

NTM: Nontuberculous mycobacteria
MTB: *Mycobacterium tuberculosis*
HIV: Human immunodeficiency virus
LJ: Lowenstein-Jensen
CDC: Centers for Disease Control and Prevention
dNTP: Deoxynucleotide triphosphate
NCBI: National Center for Biotechnology Information
